# Federated learning via over-the-air computation in IRS-assisted UAV communications

**DOI:** 10.1038/s41598-023-34292-8

**Published:** 2023-05-17

**Authors:** Ruijie Li, Li Zhu, Guoping Zhang, Hongbo Xu, Yun Chen

**Affiliations:** 1 College of Physical Science and Technology, Central China Normal University, Wuhan, 430079 China; 2 College of Intelligent Systems Science and Engineering, Hubei Minzu University, Enshi, 445000 China

**Keywords:** Engineering, Physics

## Abstract

Intelligent reflective surface (IRS) and unmanned aerial vehicle (UAV) communication are two key technologies in the sixth generation of mobile communication (6G). In this paper, IRS is equipped on UAV to form aerial IRS, which can achieve 360° panoramic full-angle reflection and flexible deployment of IRS. In order to achieve high-quality and ubiquitous network coverage under data privacy and low latency requirements, we propose an Federated learning (FL) network via Over-the-Air computation (AirComp) in IRS-assisted UAV communications. Our goal is to minimize the worst-case mean square error (MSE) by jointly optimizing the IRS phase shift, denoising factor for noise suppression, the user’s transmission power, and UAV trajectory. Optimizing and quickly adjusting the UAV position and IRS phase shift, it flexibly assists the signal transmission between users and base stations (BS). In order to solve this complex non-convex problem, we propose a low-complexity iterative algorithm, which divides the original problem into four sub-problems, respectively using the semi-definite programming (SDP) method, slack variable introduction method, successive convex approximation (SCA) method to solve each sub-problem. Through the analysis of simulation results, our proposed design scheme is obviously better than other benchmark schemes.

## Introduction

The fundamental requirements of 6G include a mobile communication network that integrates intelligence, perception, and security with Communication as the main function and achieves air-sky-earth-sea seamless coverage with human-centered and multi-network integration. UAV mainly performs communication functions at the air base network level in 6G. In order to effectively improve the quality of wireless communication networks, UAVs can be deployed to assist communication and achieve expansion at the wireless communication network level. The basic communication of the ground-based network is expanded to the space-based network. Then it can be interconnected with the space-based or sea-based satellite to achieve the 6G’s macro requirements of global coverage and scene interconnection. As a small flight device, UAV has many advantages, such as line-of-sight (LoS) channel, low cost, high mobility, and high controllability, making the 6G network more convenient. Therefore, UAV-assisted communication is an indispensable potential technology in 6G networks.

In recent years, IRS has become a research hotspot. IRS is a device consisting of a large number of low-cost passive reflection elements. Different elements of IRS can independently reflect the incident signal by controlling their amplitude and phase, with negligible power loss^1–3^. Therefore, IRS is deployed in wireless networks to provide an intelligent and reconfigurable wireless channel environment for 6G systems. However, relevant studies mainly focus on the ground IRS, including single IRS and multiple IRS. Yang et al.^4^ studied the resource allocation problem of a multi-IRS-assisted wireless communication network, which is a joint optimization problem of transmission beamforming and IRS control. The goal is to maximize energy efficiency under the user's minimum rate constraint. Mu et al.^5^ studied the basic capacity limit of IRS-assisted multi-user wireless communication systems and adopted non-orthogonal multiple access (NOMA) and orthogonal multiple access (OMA) transmission schemes for capacity realization. Ground IRS has the following characteristics: ① Ground IRS needs to be fixed on the ground building, and it is difficult to find a suitable building and installation position in practical application; ② Ground IRS can only perform 180° reflection, which requires the transmitter and receiver must be on the same side of IRS; ③ In the complex urban environment, multiple IRSs are often required to perform multiple reflections, which will lead to serious signal attenuation.

Studies have shown that where IRS is deployed can significantly affect system performance^6^. Due to its small size, portability, and low power consumption, IRS can be conveniently installed on aerial platforms such as UAVs to form aerial IRS^7,8^. Compared with traditional ground IRS, aerial IRS equipped with IRS on UAV (IRS-UAV) has the following advantages: ① UAV is located at a high altitude and increases the transmission probability of LoS by raising altitude; ② IRS-UAV has higher deployment flexibility, which can simultaneously optimize UAV trajectory and IRS phase; ③ IRS-UAV can achieve 360° panoramic full-angle reflection; ④ Even in the complex urban environment, it is often only necessary to pass through a reflection to complete the signal transmission. The above characteristics make aerial IRSs have better signal channel quality. Cai et al.^9^ studied the IRS-assisted UAV communication system to minimize the average total power consumption by jointly optimizing the UAV trajectory design and resource allocation strategy. Wei et al.^10^ studied the IRS-assisted UAV’s orthogonal frequency division multiple access (OFDMA) communication system, taking advantage of the significant beamforming gains from the IRS and the high mobility of UAV to improve the system’s sum speed. Cai et al.^9^, Wei et al.^10^ did not equip the IRS on UAVs to replace the traditional ground IRS to assist UAV communication. The research on IRS-UAV mainly adopted the traditional multiple-access method. Mao et al.^11^ studied an IRS-assisted UAV low-altitude passive aerial relay system and found that the flexibility of IRS-UAV can provide a better signal-to-noise ratio (SNR) for the system. Jiao et al.^12^ studied an IRS based UAV assisted multiple input single output (MISO) NOMA downlink communication network, first found the optimal horizontal position of the UAV, and then jointly optimized the beamforming vector and IRS phase shift matrix to maximize the data rate of the strong user in the system.

Traditional multiple access adopts the architecture of communication and computing separation. First, recover the signal of each transmitting node at the receiving end. And then calculate the objective function. This method will lead to serious energy consumption and delay. AirComp is based on “communication-computing integration” and utilizes the waveform superposition properties of signals during transmission to achieve fast data collection^13–15^. However, AirComp cannot complete all tasks, only simple tasks like summing, averaging, and finding maximum and minimum values. FL is a machine learning (ML) framework that can effectively help multiple institutions with data usage and ML modeling while meeting the requirements of user privacy protection, data security, and government regulations^16^. Only the trained model is uploaded during FL training, and the data is kept locally, solving two challenges of data privacy and communication transmission pressure in ML tasks^17^. Due to its outstanding characteristics, FL is widely used in various industries such as finance, healthcare, education, urban computing,and smart cities. Wang et al.^18^ proposed a safety-enhanced FL to predict the energy needs of electric vehicles (EVs) while considering the effectiveness of energy management in EVs and the potential risks to FL. Lian et al.^19^ proposed a decentralized, efficient and privacy-enhanced federal edge learning (FEL) system, which enables medical devices from different institutions to collaborate to train global models without exchanging raw data, and solves the privacy and security issues caused by patient data leakage for disease prediction or diagnostic models.

The FL training process generally uses the federated averaging (FedAvg) algorithm to aggregate the local models. Therefore, the combination of FL and AirComp can achieve fast data collection and complete the calculation in Communication, prevent the server from understanding the calculation process, and have better confidentiality. Yang et al.^14^ studied a fast FL global model aggregation method based on the AirComp principle to ensure the system’s strict requirements of low latency and privacy. Fu et al.^20^ studied a low-cost UAV as a mobile BS to assist the AirComp system and analyze the AirComp performance by calculating the time average MSE. There are few studies on the combination of IRS-UAV and AirComp. In order to achieve high-quality and ubiquitous network coverage under data privacy and low latency requirements, we propose an FL network via AirComp in IRS-assisted UAV communications. The main contributions of the research work are summarized as follows:

- In this paper, IRS is equipped on a UAV to form an aerial IRS, which can achieve 360° panoramic full-angle reflection and flexible deployment of IRS. Optimizing and quickly adjusting the UAV position and IRS phase shift, it flexibly assists the signal transmission between users and BS.
- This paper proposes an FL network architecture via AirComp in IRS-assisted UAV communications. With the assistance of IRS-UAV, adopting AirComp’s global aggregation method for FL, which achieves high-quality and ubiquitous network coverage under data privacy and low latency requirements.
- Our goal is to minimize the worst-case MSE by jointly optimizing the IRS phase shift $\Theta$, denoising factor for noise suppression $\eta \left[ m \right]$, the transmission power of user $p_{n} \left[ m \right]$, and UAV trajectory $q\left[ m \right]$. In order to solve this complex non-convex problem, we propose a low-complexity iterative algorithm, which divides the original problem into four sub-problems, respectively using the SDP method, slack variable introduction method, and SCA method to solve each sub-problem.

## System model

**Figure 1:**
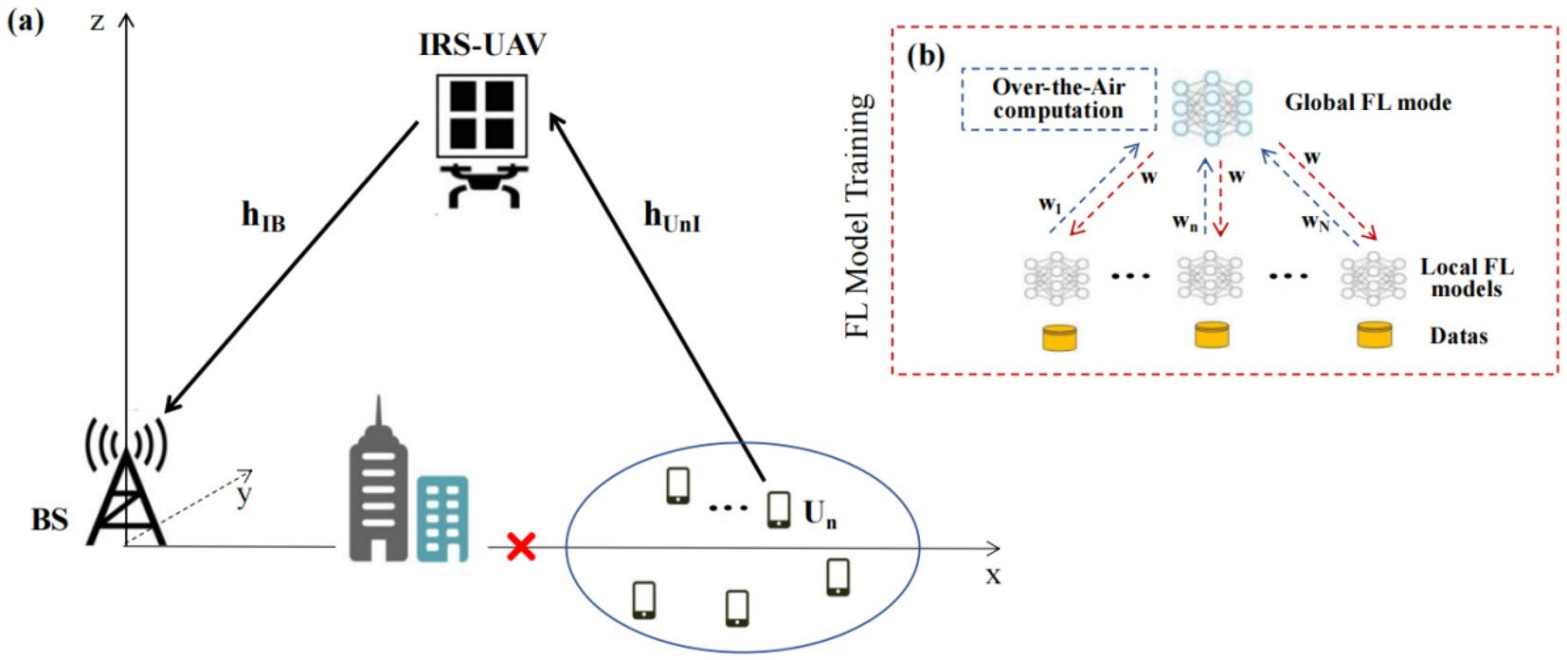
The system model diagram.

We consider an FL network via AirComp in IRS-assisted UAV communications. The system model is shown in Fig. 1a, which includes a single antenna BS, a single antenna UAV, and *N* single antenna equipment users. The user set is ${\boldsymbol{N}} = \left\{ {1,2, \ldots ,N} \right\}$. Assuming that the direct link communication between the users and BS is interrupted, an IRS-loaded rotorcraft UAV acts as a relay node to establish additional communication links between the users and BS, in which the IRS is equipped with *K* phase shift reflex elements, the phase shift reflex elements set is ${\boldsymbol{K}} = \left\{ {1,2, \ldots ,K} \right\}$. The rotorcraft UAV flies at a fixed altitude and operates from a fixed charging point. Optimizing and quickly adjusting the UAV position and IRS phase shift, it flexibly assists the signal transmission between the users and BS.

### FL model

This paper conducts FL in the IRS-assisted UAV communication network. Figure 1b shows the FL training process in detail. Suppose $w_{n} \left( {i,j} \right)$ represents the *n*-th user’s local model parameter for the *j*-th local iteration in the *i*-th global iteration, where $j \in \left\{ {1,2, \ldots ,J} \right\}$, $D_{n}$ represents the local training data set of the *n*-th user.

Step 1: **Global model broadcast.** BS broadcasts global model parameters $w\left( i \right)$ to each user via IRS-UAV.

Step 2: **Local model training.** The user combines the received $w\left( i \right)$ with the local training data set $D_{n}$ and uses the gradient descent method $w_{n} \left( {i,j} \right) = w_{n} \left( {i,j - 1} \right) - \zeta_{n} \nabla F_{n} \left( {w_{n} \left( {i,j - 1} \right)} \right)$ to train the local model parameters $w_{n} \left( {i,J} \right)$, where $\zeta$ represents the learning rate.

Step 3: **Local model upload.** The client transmits the local model parameters $w_{1} \left( {i,J} \right),w_{2} \left( {i,J} \right), \ldots ,w_{N} \left( {i,J} \right)$ to BS via IRS-UAV. The AirComp method aggregates the model during the model upload process. The BS end directly receives the aggregated model parameters $w\left( {i + 1} \right)$.

Repeat the above three steps until the model parameters converge. That is, complete the FL model training.

### Channel model

We use time-discrete technology to deal with continuous UAV trajectory design, widely used in existing UAV communication research^21^. The total time for the UAV to complete work tasks is divided into $M$ time slots. The time slot set is ${\boldsymbol{M}} = \left\{ {1,2, \ldots ,M} \right\}$, and the time occupied by each time slot is $\delta$. The BS is fixed at the horizontal ground origin $\left( {0,0} \right)$, and the BS height is represented by $h_{B}$. The rotorcraft UAV flies at a fixed height $h_{I}$, and the horizontal coordinates of the IRS-UAV at the *m*-th time slot are represented by $q\left[ m \right] = \left( {x\left[ m \right],y\left[ m \right]} \right),\,\forall m \in {\boldsymbol{M}}$. The user is on the horizontal ground, and the horizontal coordinate of the *n*-th user (U_*n*_) is represented by $r_{n} = \left( {x_{n} ,y_{n} } \right),\,\forall n \in {\boldsymbol{N}}$. Therefore, the distance between the U_*n*_ and IRS-UAV is:

1 $$d_{{U_{n} I}} \left[ m \right] = \sqrt {\left\| {q\left[ m \right] - r_{n} } \right\|^{2} + h_{I}^{2} } .$$

The distance between the IRS-UAV and BS is

2 $$d_{IB} \left[ m \right] = \sqrt {q\left[ m \right]^{2} + \left\| {h_{I} - h_{B} } \right\|^{2} } .$$

Assume that the UAV starts the task at predetermined position $q_{0}$ and ends the task at predetermined position $q_{F}$. This work ignores the flight power consumption of the UAV. The maximum velocity of the UAV during the task is represented by $V_{\max } \left( {m/s} \right)$, which must satisfy the constraint $\sqrt {\dot{x}\left[ m \right]^{2} + \dot{y}\left[ m \right]^{2} } \le V_{\max }$, where $\dot{x}\left[ m \right]$ and $\dot{y}\left[ m \right]$ are the reciprocal times of $x\left[ m \right]$ and $y\left[ m \right]$ in the *m*-th time slot, respectively. Therefore, the maneuverability constraints that the UAV should satisfy are:

3 $$\begin{aligned} & q\left[ 1 \right] = q_{0} , \\ & q\left[ M \right] = q_{F} , \\ & \left\| {q\left[ {m + 1} \right] - q\left[ m \right]} \right\| \le V_{\max } \delta ,\quad \forall m \in {\boldsymbol{M}}. \\ \end{aligned}$$

IRS-UAV is used as the air relay to transmit information, and the wireless signal is transmitted to the receiver through the air-to-ground (AtG) channel or multi-hop air channel. Compared with the ground channel, the AtG channel or air channel has a higher probability of generating LoS transmission conditions, which helps improve users' quality of service (QoS) by reducing signal attenuation. In addition, the flexibility of the UAV and IRS also enables them to relocate by sensing channel characteristics to obtain better channel conditions actively. Therefore, the channel conditions are more actively adjusted compared with fixed infrastructure communication. This paper assumes that LoS channels dominate each communication link in the system.

IRS is equipped with *K* phase shift reflex elements to establish a reflection communication link between the user and BS by regulating the phase shift of IRS. Let $h_{{U_{n} I}} \left[ m \right] \in C^{K \times 1}$ and $h_{IB} \left[ m \right] \in C^{1 \times K}$ represent the channel from U_*n*_ to IRS-UAV and from IRS-UAV to BS in the *m*-th time slot, respectively. Therefore, the cascade channel of the *m*-th time slot U_*n*_-IRS-BS is expressed as:

4 $$h_{n} \left[ m \right] = h_{IB} \left[ m \right]\Theta h_{{U_{n} I}} \left[ m \right] = \frac{{\sqrt {h_{0} } }}{{\left( {\left( {\left\| {q\left[ m \right] - r_{n} } \right\|^{2} + h_{I}^{2} } \right)\left( {q\left[ m \right]^{2} + \left\| {h_{I} - h_{B} } \right\|^{2} } \right)} \right)^{{\frac{\alpha }{4}}} }}\tilde{h}_{n} \left[ m \right].$$

where $\left| {\tilde{h}_{n} \left[ m \right]} \right| = 1$, $\Theta = diag\left( {e^{{j\theta_{1} }} ,e^{{j\theta_{2} }} \cdots ,e^{{j\theta_{K} }} } \right)$ is the diagonal phase shift matrix of IRS, $\theta_{k} \in \left[ {0,2\pi } \right],k \in {\boldsymbol{K}}$ is the phase shift of the *k*-th reflection unit, $h_{0}$ is the channel power gain at the reference distance of $d_{0} = 1$ m, and $\alpha \ge 2$ is the path loss index.

### AirComp model aggregation

The client uploads the locally calculated model parameters and sends the data to BS via AirComp with the assistance of IRS-UAV. The role of AirComp is to complete aggregation during the data upload process and send the aggregated data to BS rather than individual data per user. The expectation function received at the BS end of the *m*-th time slot is: $g\left[ m \right] = \psi \left( {\sum\nolimits_{n = 1}^{N} {\phi_{n} \left( {\omega_{n} \left[ m \right]} \right)} } \right).$ Where $\phi_{n} \left( \cdot \right)$ represents the pre-processing function of U_*n*_ and $\psi \left( \cdot \right)$ represents the post-processing function of BS.

Let $x_{n} \left[ m \right] = \phi_{n} \left( {\omega_{n} \left[ m \right]} \right)$ represent the signal uploaded by the *n*-th user. The data aggregation of FL adopts the FedAvg algorithm. Therefore, in the FL process, the expected function received by the BS end of the *m*-th time slot is:

5 $$g\left[ m \right] = \frac{1}{N}\sum\limits_{n = 1}^{N} {x_{n} \left[ m \right]} .$$

Assume that $\left\{ {x_{n} \left[ m \right],\forall n \in {\boldsymbol{N}}} \right\}$ is independent, the mean is zero, and the unit variance, that is, $E\left( {x_{n} \left[ m \right]} \right) = 0$, $E\left( {x_{n} \left[ m \right]x_{n}^{H} \left[ m \right]} \right) = 1$, $E\left( {x_{i} \left[ m \right]x_{j}^{H} \left[ m \right]} \right) = 0,\forall i \ne j.$

With the assistance of IRS-UAV, the signal received at the *m*-th time slot BS end is :

6 $$y\left[ m \right] = \sum\limits_{n = 1}^{N} {\sqrt {p_{n} \left[ m \right]} \left| {h_{IB} \left[ m \right]\Theta h_{{U_{n} I}} \left[ m \right]} \right|x_{n} \left[ m \right]} + z\left[ m \right].$$

where $p_{n} \left[ m \right]$ represents the transmitting power of the *m*-th time slot U_*n*_, $z$ represents additive white Gaussian noise (AWGN), $z\left[m\right]\sim \mathcal{C}\mathcal{N}\left(0,{\sigma }^{2}\right).$

The global FL model parameters estimated at the *m*-th slot BS are:

7 $$\hat{g}\left[ m \right] = \frac{y\left[ m \right]}{{N\sqrt {\eta \left[ m \right]} }}.$$

where $\eta \left[ m \right]$ represents the denoising factor for noise suppression.

The aggregated AirComp performance in the FedAvg algorithm is measured by MSE, which is defined as:

8 $$MSE\left[ m \right] = E\left[ {\left| {\hat{g}\left[ m \right],g\left[ m \right]} \right|^{2} } \right].$$

Substituting Eqs. (5) and (7) into Eq. (8), we can get:

9 $$\begin{aligned} MSE\left[ m \right] & = \frac{1}{{N^{2} }}E\left[ {\left| {\frac{y\left[ m \right]}{{\sqrt {\eta \left[ m \right]} }} - \sum\limits_{n = 1}^{N} {x_{n} \left[ m \right]} } \right|^{2} } \right] \\ & = \frac{1}{{N^{2} }}\left( {\sum\limits_{n = 1}^{N} {\left( {\frac{{\sqrt {p_{n} \left[ m \right]} \left| {h_{IB} \left[ m \right]\Theta h_{{U_{n} I}} \left[ m \right]} \right|}}{{\sqrt {\eta \left[ m \right]} }} - 1} \right)^{2} } + \frac{{\sigma^{2} }}{\eta \left[ m \right]}} \right). \\ \end{aligned}$$

The total time for the UAV to complete work tasks is divided into $M$ time slots. In order to meet the requirements of AirComp performance of each time slot aggregation in the FedAvg algorithm, this paper considers the worst-case MSE.

### Problem formulation

We aim to minimize the worst-case MSE by jointly optimizing the U_*n*_'s transmission power $p_{n} \left[ m \right]$, denoising factor for noise suppression $\eta \left[ m \right]$, IRS phase shift $\Theta$, and UAV trajectory $q\left[ m \right]$. According to the system model in the previous section, the optimization problem can be expressed as follows:

10 $$\begin{gathered} \begin{array}{*{20}l} {\mathop {\min }\limits_{{\Theta ,q\left[ m \right],p_{n} \left[ m \right],\eta \left[ m \right]}} } & {\mathop {\max }\limits_{\forall m} MSE\left[ m \right]} \\ {s.t.} & {C1:0 \le p_{n} \left[ m \right] \le p_{\max } ,} & {\forall n \in {\boldsymbol{N}},m \in {\boldsymbol{M}},}\\ {} & {C2:\eta \left[ m \right] \ge 0,} & {\forall m \in {\boldsymbol{M}},}\\ {} & {C3:0 \le \theta_{k} \le 2\pi ,} & {\forall k \in {\boldsymbol{K}},} \\ {} & {C4:q\left[ 1 \right] = q_{0} ,} & {} \\ {} & {C5:q\left[ M \right] = q_{F} ,} & {} \\ {} & {C6:\left\| {q\left[ {m + 1} \right] - q\left[ m \right]} \right\| \le V_{\max } \delta ,} & {\forall m \in {\boldsymbol{M}}.} \\ \end{array} \end{gathered}$$

where C1 is the transmission power constraint of the client, and $p_{\max }$ is the maximum transmission power of the client. C2 is a non-negative constraint on the denoising factor for noise suppression. C3 is the IRS phase shift constraint. C4 and C5 are the start and end positions of the UAV, respectively. C6 is the UAV flight speed constraint, and $V_{\max }$ is the maximum UAV flight speed. Due to the variable coupling between the objective function and the constraint, the optimal solution to the optimization problem (10) cannot be obtained directly.

## Proposed algorithm

In order to deal with the non-convexity of the optimization problem (10), this section proposes an iterative algorithm to decompose the problem (10) into four sub-problems. Specifically, for the optimization variables $p_{n} \left[ m \right]$, $\eta \left[ m \right]$, $\Theta$ and $q\left[ m \right]$, we optimize one of the variables and fix the others, using alternate optimization until the convergence condition is reached.

### IRS phase shift optimization

Given the optimization variables $p_{n} \left[ m \right]$, $\eta \left[ m \right]$, and $q\left[ m \right]$, problem (10) can be simplified as a sub-problem about phase optimization:

11 $$\begin{gathered} \begin{array}{*{20}l} {\mathop {\min }\limits_{\Theta } } & {\mathop {\max }\limits_{\forall m} \sum\limits_{n = 1}^{N} {\left( {\frac{{\sqrt {p_{n} \left[ m \right]} \left| {h_{IB} \left[ m \right]\Theta h_{{U_{n} I}} \left[ m \right]} \right|}}{{\sqrt {\eta \left[ m \right]} }} - 1} \right)^{2} } }\hfill \\ {s.t.} & {0 \le \theta_{k} \le 2\pi ,} & {\forall k \in {\boldsymbol{K}}.} \\ \end{array} \end{gathered}$$

Let $\beta_{k} = e^{{j\theta_{k} }} ,\,\forall k \in {\boldsymbol{K}}$, $h_{IB} \left[ m \right]\Theta h_{{U_{n} I}} \left[ m \right] = \mu_{n}^{H} \left[ m \right]\beta$. Where $\beta = \left[ {e^{{j\theta_{1} }} ,e^{{j\theta_{2} }} , \ldots ,e^{{j\theta_{K} }} } \right]^{T}$, $\mu_{n}^{H} \left[ m \right] = h_{IB} \left[ m \right]diag\left( {h_{{U_{n} I}} \left[ m \right]} \right)$. Then the optimization problem (11) is rewritten as:

12 $$\begin{gathered} \begin{array}{*{20}c} {\mathop {\min }\limits_{\beta } } & {\mathop {\max }\limits_{\forall m} \sum\limits_{n = 1}^{N} {\left( {\frac{{\sqrt {p_{n} \left[ m \right]} \left| {\mu_{n}^{H} \left[ m \right]\beta } \right|}}{{\sqrt {\eta \left[ m \right]} }} - 1} \right)^{2} } } \\ \end{array} \hfill \\ \begin{array}{*{20}c} {s.t.} & {\left| {\beta_{k} } \right|^{2} = 1,} & {\forall k \in {\boldsymbol{K}}.} \\ \end{array} \hfill \\ \end{gathered}$$

We choose to minimize an upper bound of the objective function to optimize problem (12), and transform the objective function of problem (12) as follows:

13 $$\begin{aligned} \sum\limits_{n = 1}^{N} {\left( {\frac{{\sqrt {p_{n} \left[ m \right]} \left| {\mu_{n}^{H} \left[ m \right]\beta } \right|}}{{\sqrt {\eta \left[ m \right]} }} - 1} \right)^{2} } & = \sum\limits_{n = 1}^{N} {\frac{{p_{n} \left[ m \right]}}{\eta \left[ m \right]}\mu_{n}^{H} \left[ m \right]\mu_{n} \left[ m \right]\left\| \beta \right\|^{2} } - 2R\left\{ {\sum\limits_{n = 1}^{N} {\sqrt {\frac{{p_{n} \left[ m \right]}}{\eta \left[ m \right]}} \mu_{n}^{H} \left[ m \right]\beta } } \right\} + 1 \\ & \le \lambda_{\max } \left( \Phi \right)\left\| \beta \right\|^{2} - 2R\left\{ {\beta^{H} \xi } \right\} + a \\ \end{aligned}$$

where $\Phi = \sum\limits_{n = 1}^{N} {\frac{{p_{n} \left[ m \right]}}{\eta \left[ m \right]}\mu_{n}^{H} \left[ m \right]\mu_{n} \left[ m \right]}$, $a = \tilde{\beta }^{H} \left( {\lambda_{\max } \left( \Phi \right)I - \Phi } \right)\tilde{\beta } + 1$, $\xi = \sum\limits_{n = 1}^{N} {\sqrt {\frac{{p_{n} \left[ m \right]}}{\eta \left[ m \right]}} \mu_{n} \left[ m \right]}$, $\tilde{\beta }$ is the $\beta$ solution obtained by the previous iteration in the alternating iteration algorithm. Problem (12) can be simplified as follows:

14 $$\begin{gathered} \begin{array}{*{20}c} {\mathop {\min }\limits_{\beta } } & {\lambda_{\max } \left( \Phi \right)\left\| \beta \right\|^{2} - 2R\left\{ {\beta^{H} \xi } \right\}} \\ {s.t.} & {\left| {\beta_{k} } \right|^{2} = 1,} & {\forall k \in {\boldsymbol{K}}.} \\ \end{array} \hfill \\ \end{gathered}$$

From the constraint of the problem (14), we can get $\left\| \beta \right\|^{2} = K$. When phase $\beta_{k}$ is equal to $\xi_{k}$, $R\left\{ {\beta^{H} \xi } \right\}$ gets the maximum value, where $\xi_{k}$ represents the *k*-th entrance of $\xi$. Therefore, the optimal solution to the problem (14) can be obtained as follows:

15 $$\beta^{*} = \left[ {e^{{j\arg \left( {\xi_{1} } \right)}} ,e^{{j\arg \left( {\xi_{2} } \right)}} , \ldots ,e^{{j\arg \left( {\xi_{K} } \right)}} } \right]^{T} .$$

The optimized solution $\beta^{*}$ is a closed form, which is easier to implement and has less computational complexity than the semi definite relaxation (SDR) method.

### Denoising factor optimization

Using the optimized $\Theta$ and the given optimization variables $p_{n} \left[ m \right]$ and $q\left[ m \right]$, problem (10) can be simplified to a sub-problem about the denoising factor optimization:

16 $$\begin{gathered} \begin{array}{*{20}l} {\mathop {\min }\limits_{\eta \left[ m \right]} } & {\mathop {\max }\limits_{\forall m} \sum\limits_{n = 1}^{N} {\left( {\frac{{\sqrt {p_{n} \left[ m \right]} \left| {h_{n} \left[ m \right]} \right|}}{{\sqrt {\eta \left[ m \right]} }} - 1} \right)^{2} } + \frac{{\sigma^{2} }}{\eta \left[ m \right]}} \\ {s.t.} & {\eta \left[ m \right] \ge 0,} & {\forall m \in {\boldsymbol{M}}.} \\ \end{array} \hfill \\ \end{gathered}$$

Let $\gamma \left[ m \right] = \frac{1}{{\sqrt {\eta \left[ m \right]} }}$, the optimization problem (16) is rewritten as:

17 $$\begin{gathered} \begin{array}{*{20}l} {\mathop {\min }\limits_{\gamma \left[ m \right]} } & {\mathop {\max }\limits_{\forall m} \sum\limits_{n = 1}^{N} {\left( {\sqrt {p_{n} \left[ m \right]} \left| {h_{n} \left[ m \right]} \right|\gamma \left[ m \right] - 1} \right)^{2} } + \sigma^{2} \gamma \left[ m \right]^{2} } \\ {s.t.} & {\gamma \left[ m \right] \ge 0,} & {\forall m \in {\boldsymbol{M}}.} \\ \end{array} \hfill \\ \end{gathered}$$

The objective function in problem (17) is a quadratic function of one variable with respect to variable $\gamma \left[ m \right]$. By introducing variable $\chi$, problem (17) can be transformed into:

18 $$\begin{gathered} \begin{array}{*{20}l} {\mathop {\min }\limits_{\gamma \left[ m \right],\chi } } & \chi \\ {s.t.} & {\sum\limits_{n = 1}^{N} {\left( {\sqrt {p_{n} \left[ m \right]} \left| {h_{n} \left[ m \right]} \right|\gamma \left[ m \right] - 1} \right)^{2} } + \sigma^{2} \gamma \left[ m \right]^{2} \le \chi ,} & {\forall n \in {\boldsymbol{N}},m \in {\boldsymbol{M}},} \\ {} & {\gamma \left[ m \right] \ge 0,} & {\forall m \in {\boldsymbol{M}}.} \\ \end{array} \hfill \\ \end{gathered}$$

Problem (18) is co-convex with respect to the optimization variables $\gamma \left[ m \right]$ and $\chi$, which is a quadratic constrained quadratic programming (QCQP) problem. Therefore, Problem (18) can be solved directly by the convex optimization toolkit.

### Transmission power optimization

Using the optimized $\Theta$, $\eta \left[ m \right]$, and the given optimization variable $q\left[ m \right]$, problem (10) can be simplified to a sub-problem about the transmitted power optimization:

19 $$\begin{gathered} \begin{array}{*{20}l} {\mathop {\min }\limits_{{p_{n} \left[ m \right]}} } & {\mathop {\max }\limits_{\forall m} \sum\limits_{n = 1}^{N} {\left( {\frac{{\sqrt {p_{n} \left[ m \right]} \left| {h_{n} \left[ m \right]} \right|}}{{\sqrt {\eta \left[ m \right]} }} - 1} \right)^{2} } } \\ {s.t.} & {0 \le p_{n} \left[ m \right] \le p_{\max } ,} & {\forall n \in {\boldsymbol{N}},m \in {\boldsymbol{M}}.} \\ \end{array} \hfill \\ \end{gathered}$$

By introducing variable $w$, problem (19) can be transformed into:

20 $$\begin{gathered} \begin{array}{*{20}l} {\mathop {\min }\limits_{{p_{n} \left[ m \right],w}} } & w \\ \end{array} \hfill \\ \begin{array}{*{20}c} {s.t.} & {\sum\limits_{n = 1}^{N} {\left( {\frac{{\sqrt {p_{n} \left[ m \right]} \left| {h_{n} \left[ m \right]} \right|}}{{\sqrt {\eta \left[ m \right]} }} - 1} \right)^{2} } \le w,} & {\forall n \in {\boldsymbol{N}},m \in {\boldsymbol{M}},} \\ {} & {0 \le p_{n} \left[ m \right] \le p_{\max } ,} & {\forall n \in {\boldsymbol{N}},m \in {\boldsymbol{M}}} \\ \end{array} \hfill \\ \end{gathered}$$

Problem (20) is co-convex with respect to the optimization variables $p_{n} \left[ m \right]$ and $w$, which is a QCQP problem. Therefore, Problem (20) can be solved directly by the convex optimization toolkit.

### UAV trajectory optimization

Using the optimized $\Theta$, $\eta \left[ m \right]$ and $p_{n} \left[ m \right]$ to optimize the variable $q\left[ m \right]$, Problem (10) can be simplified to a sub-problem about the UAV trajectory optimization:

21 $$\begin{gathered} \begin{array}{*{20}l} {\mathop {\min }\limits_{q\left[ m \right]} } & {\mathop {\max }\limits_{\forall m} \sum\limits_{n = 1}^{N} {\left( {\frac{{\sqrt {p_{n} \left[ m \right]} \sqrt {h_{0} } }}{{\sqrt {\eta \left[ m \right]} \left( {\left( {\left\| {q\left[ m \right] - r_{n} } \right\|^{2} + h_{I}^{2} } \right)\left( {q\left[ m \right]^{2} + \left\| {h_{I} - h_{B} } \right\|^{2} } \right)} \right)^{{\frac{\alpha }{4}}} }} - 1} \right)^{2} } } \\ {s.t.} & {C4,C5,C6.} \\ \end{array} \hfill \\ \end{gathered}$$

Let $u_{n} \left[ m \right] = \frac{{p_{n} \left[ m \right]h_{0} }}{{\eta \left[ m \right]\left( {\left( {\left\| {q\left[ m \right] - r_{n} } \right\|^{2} + h_{I}^{2} } \right)\left( {q\left[ m \right]^{2} + \left\| {h_{I} - h_{B} } \right\|^{2} } \right)} \right)^{{\frac{\alpha }{2}}} }}$, $v_{n} \left[ m \right] = \frac{{2\sqrt {p_{n} \left[ m \right]} \sqrt {h_{0} } }}{{\sqrt {\eta \left[ m \right]} \left( {\left( {\left\| {q\left[ m \right] - r_{n} } \right\|^{2} + h_{I}^{2} } \right)\left( {q\left[ m \right]^{2} + \left\| {h_{I} - h_{B} } \right\|^{2} } \right)} \right)^{{\frac{\alpha }{4}}} }}$. Then the optimization problem (21) is rewritten as:

22 $$\begin{gathered} \begin{array}{*{20}l} {\mathop {\min }\limits_{q\left[ m \right]} } & {\mathop {\max }\limits_{\forall m} \sum\limits_{n = 1}^{N} {\left( {u_{n} \left[ m \right] - v_{n} \left[ m \right]} \right)} } \\ {s.t.} & {C4,C5,C6.} \\ \end{array} \hfill \\ \end{gathered}$$

Since $u_{n} \left[ m \right]$ and $v_{n} \left[ m \right]$ are neither convex nor concave with respect to variable $q\left[ m \right]$, problem (22) is a non-convex problem. For $u_{n} \left[ m \right]$ is a convex function of variable $\sqrt {\left( {\left\| {q\left[ m \right] - r_{n} } \right\|^{2} + h_{I}^{2} } \right)\left( {q\left[ m \right]^{2} + \left\| {h_{I} - h_{B} } \right\|^{2} } \right)}$, the relaxation variable $D_{n} \left[ m \right]$ is introduced to solve the non-convexity of the problem (22).

23 $$\begin{gathered} \begin{array}{*{20}l} {\mathop {\min }\limits_{{q\left[ m \right],D_{n} \left[ m \right]}} } & {\sum\limits_{n = 1}^{N} {\frac{{p_{n} \left[ m \right]h_{0} }}{{\eta \left[ m \right]D_{n} \left[ m \right]^{\alpha } }}} - \mathop {\max }\limits_{\forall m} \sum\limits_{n = 1}^{N} {v_{n} \left[ m \right]} } \\ {s.t.} & {C4,C5,C6,} \\ \end{array} \hfill \\ \begin{array}{*{20}c} {} & {D_{n} \left[ m \right] \le \sqrt {\left( {\left\| {q\left[ m \right] - r_{n} } \right\|^{2} + h_{I}^{2} } \right)\left( {q\left[ m \right]^{2} + \left\| {h_{I} - h_{B} } \right\|^{2} } \right)} ,} & {\forall n \in {\boldsymbol{N}},m \in {\boldsymbol{M}},} \\ {} & {D_{n} \left[ m \right] \ge 0,} & {\forall n \in {\boldsymbol{N}},m \in {\boldsymbol{M}}.} \\ \end{array} \hfill \\ \end{gathered}$$

The objective function and constraint conditions of problem (23) are still non-convex, so solving problem (23) has a significant challenge. Using the SCA algorithm to approximate the non-convexity of the problem (23). Based on the first-order Taylor expansion of $v_{n} \left[ m \right]$ and $\sqrt {\left( {\left\| {q\left[ m \right] - r_{n} } \right\|^{2} + h_{I}^{2} } \right)\left( {q\left[ m \right]^{2} + \left\| {h_{I} - h_{B} } \right\|^{2} } \right)}$ in the *l*-th iteration at $\left\{ {q^{l} \left[ m \right],\forall m \in {\boldsymbol{M}}} \right\}$, we can obtain its global lower bound:

24 $$v_{n} \left[ m \right] \ge v_{n}^{l} \left[ m \right] - \frac{{\alpha \sqrt {p_{n} \left[ m \right]} \sqrt {h_{0} } }}{{\sqrt {\eta \left[ m \right]} Q_{n}^{l} \left[ m \right]^{{\frac{\alpha + 2}{2}}} }}\left( {Q_{n} \left[ m \right] - Q_{n}^{l} \left[ m \right]} \right) = \tilde{v}_{n}^{l} \left[ m \right],$$

25 $$\begin{aligned} Q_{n} \left[ m \right]& \ge Q_{n}^{l} \left[ m \right] + \frac{{\left( {q\left[ m \right] - q^{l} \left[ m \right]} \right)^{T} \left( {\left( {q^{l} \left[ m \right] - \gamma_{n} } \right)\left( {q^{l} \left[ m \right]^{2} + \left\| {h_{I} - h_{B} } \right\|^{2} } \right) + q^{l} \left[ m \right]\left( {\left\| {q^{l} \left[ m \right] - r_{n} } \right\|^{2} + h_{I}^{2} } \right)} \right)}}{{\sqrt {Q_{n}^{l} \left[ m \right]} }} \\ &= \tilde{Q}_{n}^{l} \left[ m \right]. \\ \end{aligned}$$

where $Q_{n} \left[ m \right] = \sqrt {\left( {\left\| {q\left[ m \right] - r_{n} } \right\|^{2} + h_{I}^{2} } \right)\left( {q\left[ m \right]^{2} + \left\| {h_{I} - h_{B} } \right\|^{2} } \right)}$. Substitute the lower bound in Eqs. (24) and (25) into the problem (23). For any given local point $\left\{ {q^{l} \left[ m \right],\forall m \in {\boldsymbol{M}}} \right\}$, problem (23) is approximately transformed into the following form:

26 $$\begin{gathered} \begin{array}{*{20}l} {\mathop {\min }\limits_{{q\left[ m \right],D_{n} \left[ m \right]}} } & {\sum\limits_{n = 1}^{N} {\left( {\frac{{p_{n} \left[ m \right]h_{0} }}{{\eta \left[ m \right]D_{n} \left[ m \right]^{\alpha } }} - \tilde{v}_{n}^{l} \left[ m \right]} \right)} } \\ {s.t.} & {C4,C5,C6,} \\ {} & {D_{n} \left[ m \right] \le \tilde{Q}_{n}^{l} \left[ m \right],} & {\forall n \in {\boldsymbol{N}},m \in {\boldsymbol{M}},} \\ {} & {D_{n} \left[ m \right] \ge 0,} & {\forall n \in {\boldsymbol{N}},m \in {\boldsymbol{M}}.} \\ \end{array} \hfill \\ \end{gathered}$$

Problem (26) is co-convex with respect to the optimization variables $q\left[ m \right]$ and $D_{n} \left[ m \right]$, which is a QCQP problem. Therefore, problem (26) can be solved directly by the convex optimization toolkit. See algorithm 1 for the specific algorithm steps.
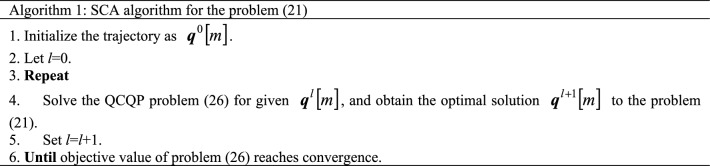


The transformed problems (18), (20), and (26) are convex and solved by the convex optimization solver CVX. The optimal solutions to problems (16), (19), and (21) are obtained, respectively. The closed solution to the problem (11) is obtained by transformation. Then the optimal solution to the optimization problem (10) is finally obtained through the iterative algorithm. In summary, the iterative algorithm of the proposed problem (10) can be presented in algorithm 2.
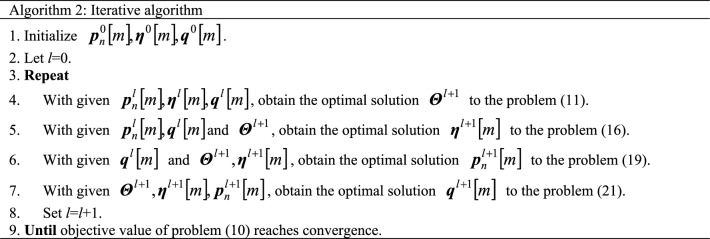


### Complexity analysis

This paper proposes a low-complexity iterative algorithm, which divides the optimization problem (10) into four sub-problems, namely, problem (11), problem (16), problem (19), and problem (21). The algorithm is solved by algorithm 2. In each iteration, problem (11) obtains a closed solution; Problem (16) and problem (19) are transformed into QCQP problem (18) and problem (20) respectively by introducing relaxation variables, with the complexity of $O(N^{2.5} M^{2.5} )$. Problem (21) is transformed into QCQP problem (26) through SCA approximation, with complexity of $O(N^{2.5} M^{2.5} )$. Therefore, the computational complexity of the algorithm is $O(N^{2.5} M^{2.5} )$.

## Results analysis

In this section, we analyze the performance of the proposed algorithm through simulation results. An IRS-loaded UAV flies at an altitude of 100 m, and 12 users are randomly distributed in a circular area with (150,0) as the center and 50 m as the radius. Assume that the channels between the user and IRS-UAV and between the IRS-UAV and BS are both LoS. For large-scale fading, the path loss index $\alpha = 2$; For small-scale fading, Rice fading channel is selected. Table 1 shows other relevant parameter settings.

**Table 1:** Parameter settings.

Parameter	Value
UAV initial position, $q_{0}$	(− 200,0)
UAV destination position, $q_{F}$	(500,0)
UAV height, $h_{I}$	100 m
BS height, $h_{B}$	20 m
Time slot length, $\delta$	0.2 s
UAV maximum flight speed, $V_{\max }$	30 m/s
Channel power gain, $h_{0}$	− 40 dB
Learning rate, $\zeta$	0.001
Path loss index, $\alpha$	2
Noise power, $\sigma^{2}$	− 110 dBm

**Figure 2:**
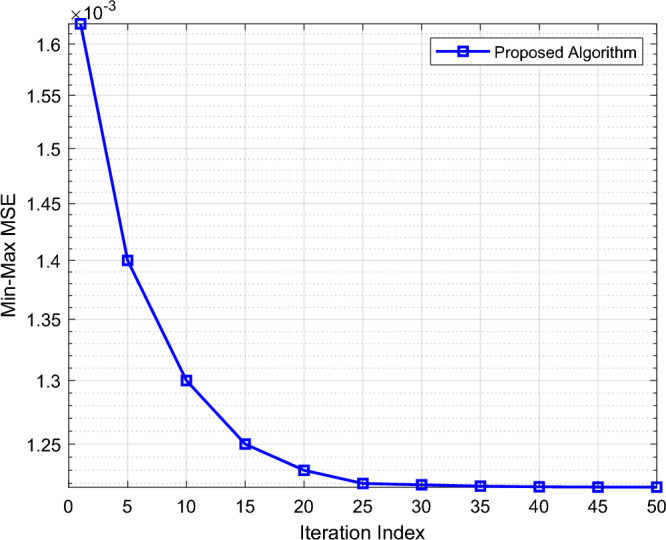
Convergence analysis.

In Fig. 2, we analyze the convergence of the proposed algorithm. It can be seen from Fig. 2 that the minimum value of MSE in the worst case (Min–Max MSE) decreases rapidly first and then reaches convergence gradually and steadily with the increase of the iteration numbers, which proves the effectiveness of the proposed algorithm jointly optimizing the U_*n*_'s transmission power $p_{n} \left[ m \right]$, denoising factor for noise suppression $\eta \left[ m \right]$, IRS phase shift $\Theta$, and UAV trajectory $q\left[ m \right]$.

**Figure 3:**
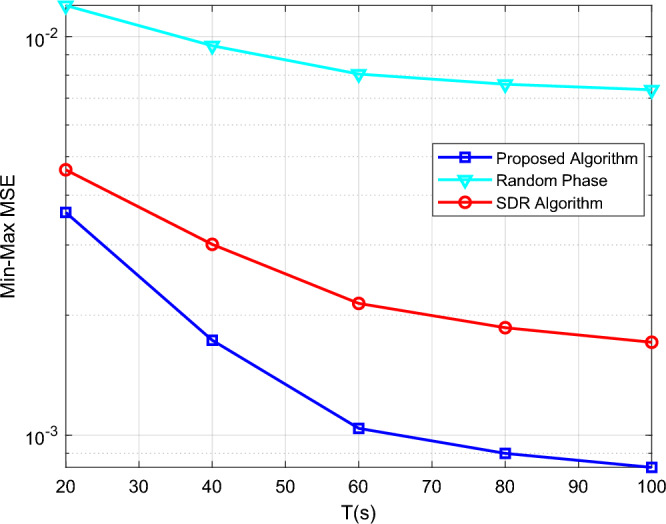
The Min–Max MSE versus mission time.

**Figure 4:**
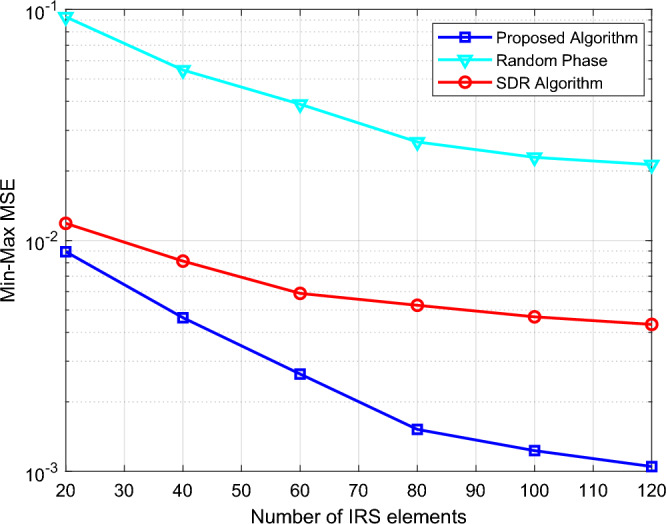
The Min–Max MSE versus the number of IRS elements.

To analyze the performance of the proposed algorithm, we design three different methods to compare the Min–Max MSE: ① SDP algorithm; ② Random phase; ③SDR algorithm. Figure 3 shows the relationship between the Min–Max MSE and task time, and the Min–Max MSE of all schemes decreases significantly with the increase in task time. The performance of our proposed algorithm is significantly better than that of the random phase and SDR algorithm under different task times. Figure 4 shows the relationship between the Min–Max MSE and the number of IRS elements *K*. With the increase of *K*, the Min–Max MSE in all cases decreases significantly, indicating that the increase in the number of IRS elements will promote the performance of the system. However, in different optimization algorithms, the optimized phase algorithm at *K* = 50 is much superior to the random phase algorithm at *K* = 100. This indicates the importance of the optimized phase in practical applications.

**Figure 5:**
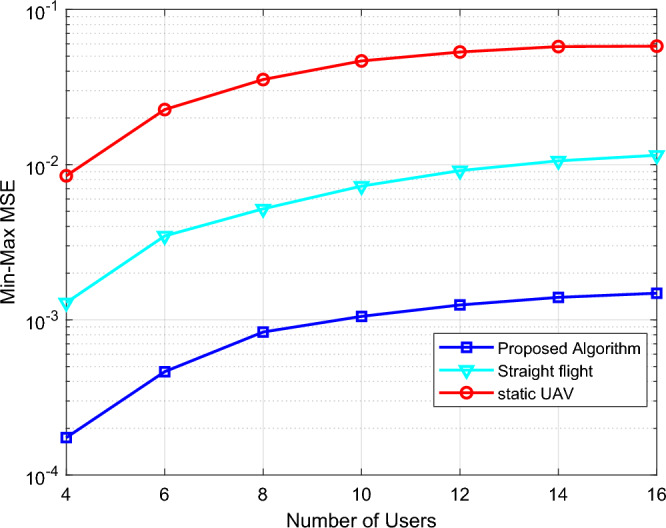
The Min–Max MSE versus the number of users.

Figure 5 shows the relationship between the Min–Max MSE and the number of users *N*, and compares three optimization algorithms: ① Optimize the UAV trajectory; ② Straight flight: the UAV flies from $q_{0}$ to $q_{F}$ in a straight line at 100 m; ③ Static UAV: the UAV is deployed at (150,0,100) and remains stationary, which is equivalent to fixing IRS at a certain height. It can be seen from Fig. 5 that the Min–Max MSE in all cases increases significantly with the increase of N, indicating that the increase in users will reduce the system performance. As can be observed in both Figs. 4 and 5, our proposed design scheme is obviously better than the other two benchmark schemes. The performance gains are more significant as the number of IRS elements increases or the number of users decreases. This is because UAV has many advantages such as LoS channel, low cost, high mobility, and high controllability. By optimizing the trajectory of UAV and the phase of IRS, the IRS-UAV can achieve an optimal balance with the BS and users, and enhance the channels of all links, thus improving the Aircomp performance of the system.

## Conclusion

FL solves two challenges of data privacy and communication transmission pressure in ML tasks. AirComp provides a promising method for the ultra-fast model aggregation of FL. The combination of FL and AirComp can not only achieve fast data collection, but also complete the calculation in communication can avoid the server from understanding the calculation process, and has better confidentiality. This paper proposes a new three-dimensional network architecture: FL network architecture via AirComp in IRS-assisted UAV communications. With the assistance of IRS-UAV, we are adopting AirComp’s global aggregation method for FL, which achieves high-quality and ubiquitous network coverage under data privacy and low latency requirements. Due to the variable coupling between the objective function and the constraint, the optimal solution cannot be obtained directly. We propose a low-complexity iterative algorithm to decompose the original problem into four sub-problems. Specifically, for the optimization variables $p_{n} \left[ m \right]$, $\eta \left[ m \right]$, $\Theta$ and $q\left[ m \right]$, we optimize one of the variables and fix the others, using alternate optimization until the convergence condition is reached.

## Data Availability

The data used to support the findings of this study are available from the corresponding author on reasonable request.
